# Development of a behaviour change intervention using a theory-based approach, Behaviour Centred Design, to increase nurses’ hand hygiene compliance in the US hospitals

**DOI:** 10.1186/s43058-021-00124-x

**Published:** 2021-02-18

**Authors:** Madeline Sands, Robert Aunger

**Affiliations:** 1 Department of Disease Control, London School of Hygiene and Tropical Medicine, London, UK; 2 University of Arizona College of Medicine, Tucson, Arizona USA

**Keywords:** Behaviour change, Intervention design, Intervention development, Hand hygiene, Hand hygiene compliance, Healthcare workers, Behaviour change techniques, Behaviour Centred Design (BCD)

## Abstract

**Background:**

A behaviour change campaign is unlikely to be effective if its intervention is not carefully designed. While numerous frameworks are widely used to develop and evaluate interventions, the steps detailing how to create an intervention are not as clear because the process of linking behaviour analysis to the intervention design is seldom discussed. We document the application of the Behaviour Centred Design (BCD) approach to the development of an intervention to improve hand hygiene (HH) rates among nurses’ hospital units in the USA.

**Methods:**

Intervention development is divided into the first three steps of the BCD approach: Assess, Build, and Create. The Assess step centres on understanding the target behaviour. The Build step expands the knowledge of the target behaviour and population through formative research which leads to a creative brief that explains the focus of the intervention. In the Create step, the creative brief guides the intervention design.

**Results:**

Drawing from the main findings of the Asses and Build steps, a focal insight was developed positing that nurses can rediscover the meaning and purpose of their role as a nurse and thus as a caregiver by practicing HH; in the process of cleaning their hands, nurses are living up to their ideal nurse-self. The focal insight was linked linguistically into a theory and change. The outcome was a simple intervention, called the *Mainspring Intervention*, which consisted of three major parts: a self-affirmation exercise to reduce defensiveness, a message that challenged nurses’ perceptions about their HH practice, and an implementation intention activity to help nurses link HH behaviour to a cue.

**Conclusions:**

We detailed the creation of an original HH intervention that used the BCD approach. The intervention is relatively simple compared to most HH initiatives in the literature, both in terms of having relatively few components to the intervention and relatively easy field implementation. This intervention will allow us to test how specific psychological processes contribute to the problem of low HH rates, how our proposed intervention changes these processes in the hospital setting, and how the expected change in nurses’ cognition transforms over time because of the intervention.

**Supplementary Information:**

The online version contains supplementary material available at 10.1186/s43058-021-00124-x.

Contributions to the literature

- We describe and document the BCD approach to intervention development, and in so doing, set forth systematic procedures for designing and refining techniques to be utilized in behaviour change interventions regarding healthcare workers in hospital settings.
- We detail how to identify and develop creative insights into actual intervention materials through linking behaviour analysis to the design of an intervention.
- The final product was the creation of an original HH behaviour change intervention, called a ‘wise’ intervention, which has not previously been used—to our knowledge—to improve healthcare workers’ hand hygiene behaviour.

## Introduction

Healthcare-associated infections (HAIs) are a global patient safety concern [1, 2]. They are associated with an increased attributable mortality, length of stay, and healthcare costs incurred by patients and healthcare facilities [3]. The causes of HAIs vary, but all can be attributed to health systems and processes of care provision. Hand hygiene (HH) is recognized as the single most important measure for preventing the spread of HAIs [2, 4, 5]. And yet, hand hygiene compliance (HHC) rates are known to be suboptimal despite the abundance of evidence that HH prevents HAIs and the increased pressure from regulatory bodies worldwide to improve compliance [6, 7]. Over the past several decades, numerous campaigns promoting HH have been launched [8]. Many of the improvement strategies to date take a multimodal approach to behaviour change including provision of alcohol-based hand rub (ABHR) and soap at point of care, training and education, reminders, administration support, and measurement of compliances rates [6]. However, improving HHC and sustaining this behavioural change remains a significant challenge [2, 8–11]. Not only is HH a complex behaviour with numerous facilitators and barriers, but it is a behaviour that occurs in a complicated and sometimes unpredictable healthcare environment [7]. Thus, a HH behaviour change campaign is unlikely to be effective if its intervention is not carefully designed.

There are a myriad of approaches to intervention development, including but not limited to the MRC Framework for developing and evaluation complex interventions, the Behaviour Change Wheel (BCW), intervention mapping, Matrix Assisting Practioner’s Intervention Planning Tool (MAP-IT), Theoretical Domains Framework (TDF), and Six Essential Steps for Quality Intervention Development (6SQUID) [12]. While each approach is grounded in a different theory or philosophy, there are similarities in how researchers are guided through the various stages of intervention development, such as agreeing on a problem, researching that problem, implementing a solution, and evaluating its effectiveness [12]. However, an issue contributing the shortcomings in intervention design is the lack of agreed practical ‘how to’ guidance for creating interventions [13]. In addition, intervention design steps detailing how to identify and develop creative insights into actual intervention materials are not as clear because the process of linking behaviour analysis to the design of an intervention is seldom discussed.

In this paper, we describe the process of designing an intervention to improve HHC among nurses in the US hospitals. We chose to promote HHC among nurses as they are among the HCWs who spend the majority of their time in direct patient contact and therefore have a greater number of opportunities to perform HH [14, 15].

**Fig. 1:**
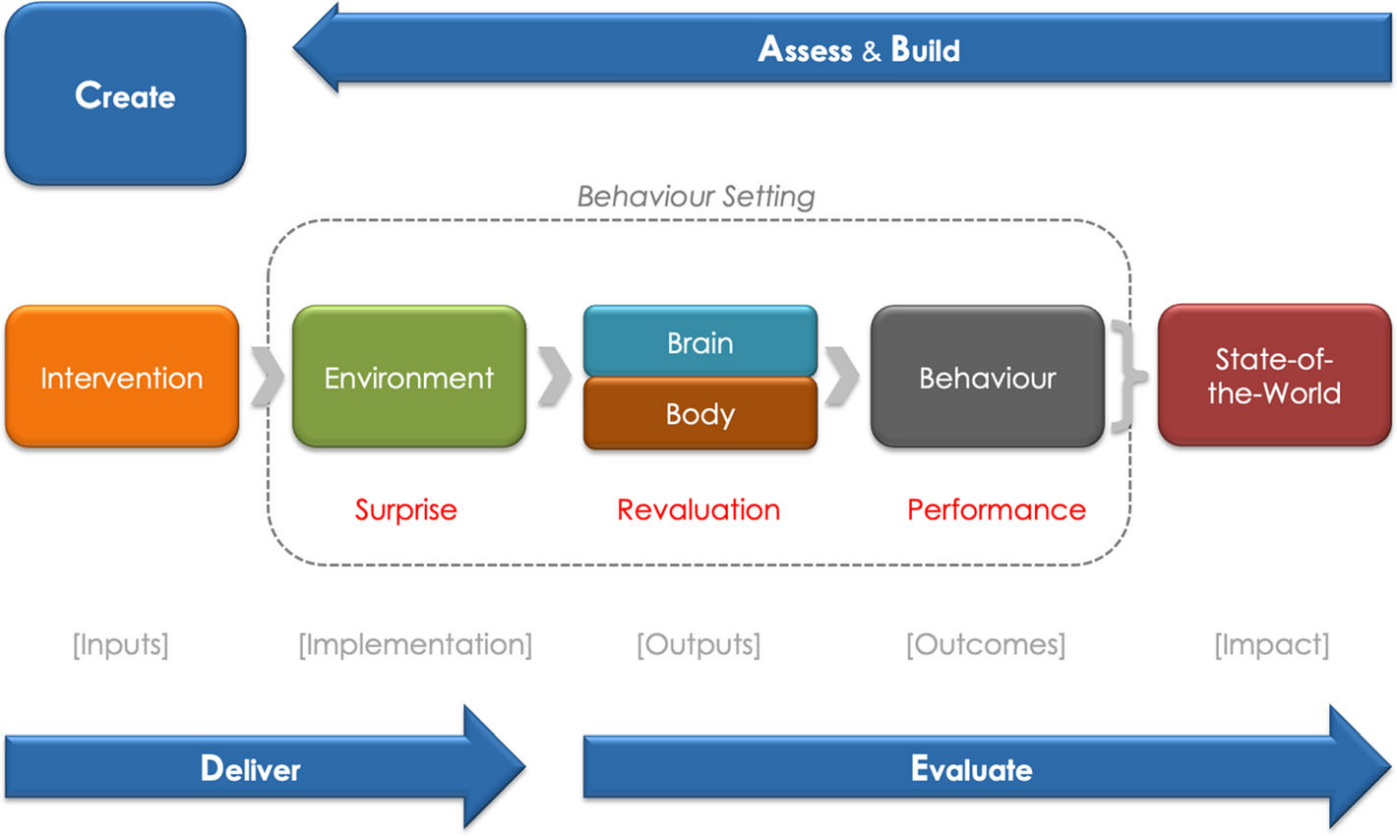
Behaviour Centred Design (BCD). Reprinted from Aunger and Curtis, 2016 with permission [16]. BCD presents a systematic way to develop a program through five steps

In designing this intervention, we used an approach called Behaviour Centred Design (BCD), which is a systematic way to develop a program through five steps (Fig. 1) [16]. The first step—Assess—is concerned with setting out the scope of the intervention and identifying what is known about the target behaviour. This serves as the basis for the following step—Build—which seeks to fill knowledge gaps essential in the development of the theory of change. Determining the Theory of Change allows for the formation of potential intervention themes, components, scope, and sequences which are necessary for generating the intervention itself in the Create step. The intervention is subsequently implemented in the Deliver step and assessed in the Evaluation step. Intervention design occurs throughout the Assess, Build, and Create steps. The basic premise behind BCD’s design process is that the settings where the target behaviour occurs must be disrupted to force revaluation of the desired behavioural option, which then causes people to perform that behaviour. Thus, interventions are tasked with creating surprising new stimuli that run counter to the brain’s predictions about the consequences of performing the target behaviour. By doing so, the brain is forced to reconsider its expectations of the value of performing different options, resulting in a trial of the target behaviour. While BCD draws from other approaches and theories, it has the following strengths:

- BCD provides both a theory of change for behaviour as well as a process for designing and evaluating interventions.
- Its theory of change is unusual in that it incorporates reinforcement learning theory, the evolution of behavioural control, the evolved structure of human motivation, and a revised version of behaviour settings theory.
- Its design process highlights the importance of formative research, which is often overlooked in intervention design approaches. The process also integrates creative insight-generation processes from Design Thinking.
- It focuses on behaviour in its physical, social, biological and temporal context.

In this paper, we describe, document, and explicate the applied BCD intervention development framework using this case study, and in so doing, set forth systematic procedures for designing and refining proven techniques to be utilized in behaviour change interventions for HCWs in hospital settings. In describing our intervention, we use the taxonomy of behaviour change techniques (BCTs) developed by *Michie et al*. (2013) [17] to label the active components. A BCT is defined as ‘an observable, replicable and irreducible component of an intervention designed to alter or redirect causal processes that regulate beahviour’. [17] BCTs thus provide the necessary linkage between an individual’s exposure to implemented elements of the intervention and the psychological consequences of that exposure.

This paper focuses on linking BCD’s Assess and Build steps with the Create step, thereby illustrating the process behind the design and development of the intervention that is not as clearly documented with other approaches.

## Methods

The development of the intervention is divided into three steps: Assess, Build, and Create. Each of these steps has a unique process and is dependent on preceding steps. Here, we describe the processes that are undertaken for each step; the results of each step follow in the subsequent section, with discussion afterwards.

### Assess

The Assess step is separated into two phases: background review and framing. The background review seeks to understand the target behaviour of HH in its context. The purpose of the framing process is to define what is within the scope of the intervention and within the means of the behaviour change practitioners.

#### Background review

A systematic review is completed to assemble existing knowledge on HH interventions targeting nurses in hospitals. The findings should provide insight into the current state of nursing HH interventions by describing how interventions have changed, detailing what present-day interventions look like, and identifying areas for improvement in intervention design.

For this study, a systematic review was performed guided by the PRISMA protocol to evaluate the use of behaviour change techniques in interventions aimed at promoting HH practices among nurses in the hospital setting. Multiple databases and reference lists were searched.

#### Framing workshop

**Fig. 2:**
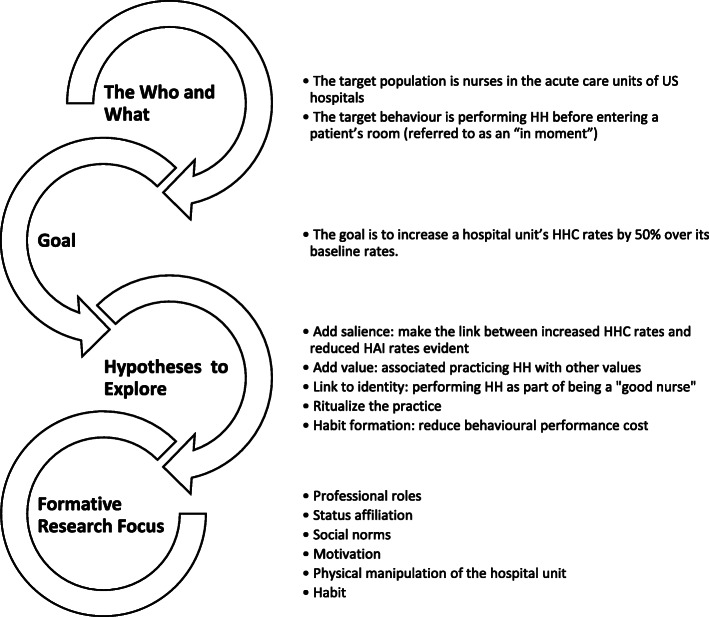
Insight and focus from framing workshop. The workshop ends with a framing statement that serves as the foundation on which the rest of the project is built

Here stakeholders and experts participate in the Framing Workshop to discuss the target behaviour and factors identified from the general survey of the literature, to agree on the aim of the intervention, and to outline the various constraints surrounding the intervention design. These stakeholders and experts will become the core group guiding the research project. The workshop ends with a framing statement that serves as the foundation on which the rest of the project is built (Fig. 2). By defining the scope of the project and compiling an extensive evidence base, the team can pinpoint what still needs to be learned and tests for potential levers of change in the Build step.

For this study, a framing workshop was held in November 2015 in the USA with stakeholders (employees of the Project Funder) and experts (nurses and Infectious Disease directors from local hospitals). The research team—primarily RA and MHS—was present to lead the workshop. During the framing workshop, the Project Funder discussed the company and its purpose. The research team discussed BCD and how it would be implemented in this project. The experts discussed HH behaviour with the group focusing on the importance of HH to control HAIs, HH recommendations, HHC rates among HCWs, factors affecting HHC rates, and current HH initiatives. The Project Manager (an employee of the Project Funder) discussed the purpose and the intended goals of the project as set forth by the Project Funder. After presentations and discussions, the team engaged in a brainstorm and affinity diagram exercise to develop the framing statement. We sought to answer the who, what, when, why, and how of the intervention. For each question, individuals wrote their ideas or responses on sticky notes. The notes were attached to the wall, grouped together under the question they were trying to answer. Similar ideas within these groups were clustered together. The team then discussed each group in length until a clear answer emerged for each question and consensus was achieved. The result was a framing statement that would guide the rest of the research project.

### Build

This stage expands the knowledge of the target behaviour and population. This involves conducting formative research that seeks to address the questions left unanswered during the framing process and literature reviews while also exploring hypotheses developed in the Assess stage.

#### Formative research

Formative research is conducted with the objective to evaluate the behavioural change potential of factors identified from the Assess stage. In this study, a questionnaire was developed to assess the potential impact of several unexamined factors in the HH literature; the questionnaire was delivered online to a panel of acute care nurses working in the US hospitals.

#### Design workshop

**Fig. 3:**
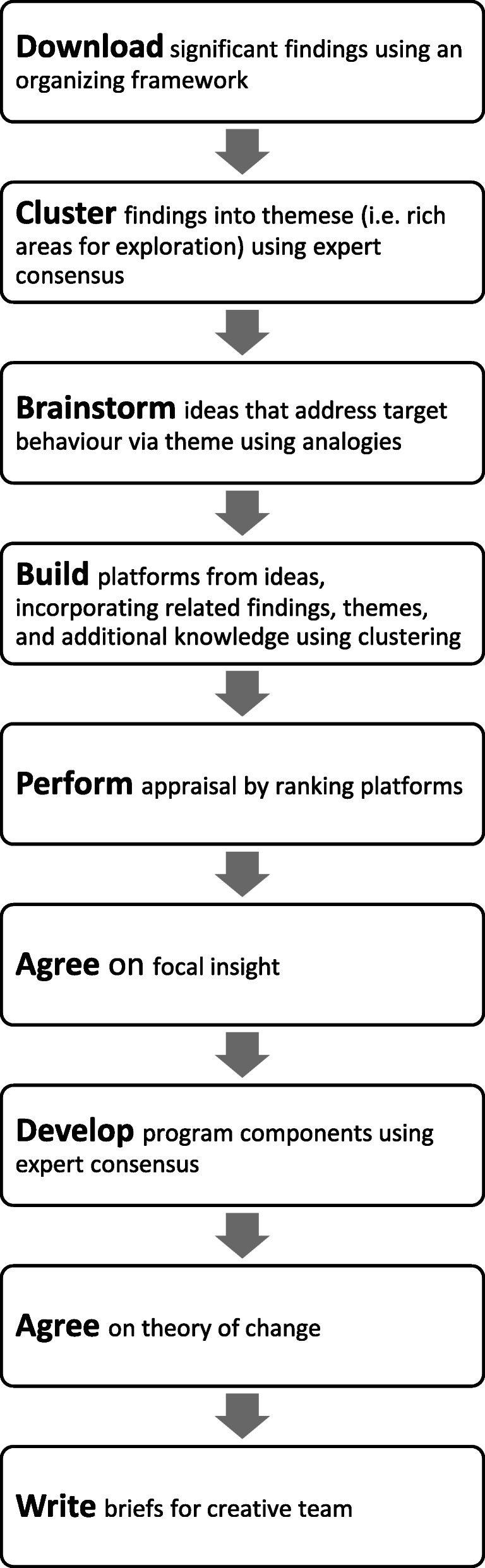
BCD’s design process for producing a focal insight. The design process can be described as a sequence of nine phases, which starts from analysing the findings from the field and concludes with a creative brief that explains the single focus of the intervention

Next, a Design Workshop is held. A team is collected together with a variety of backgrounds, expertise, and degrees of familiarity with the problem at hand. This includes the core group that participated in the framing process workshop as well as members from academia, marketing, and members of the target population. At this workshop, the findings from the formative research are presented and then converted into a Theory of Change for the intervention using BCD’s creative design process. The design process is described as a sequence of nine phases, starting with analysing the findings from the field and concluding with a creative brief that explains the single focus of the intervention (Fig. 3); this is a form of affinity mapping or thematic analysis.

**Fig. 4:**
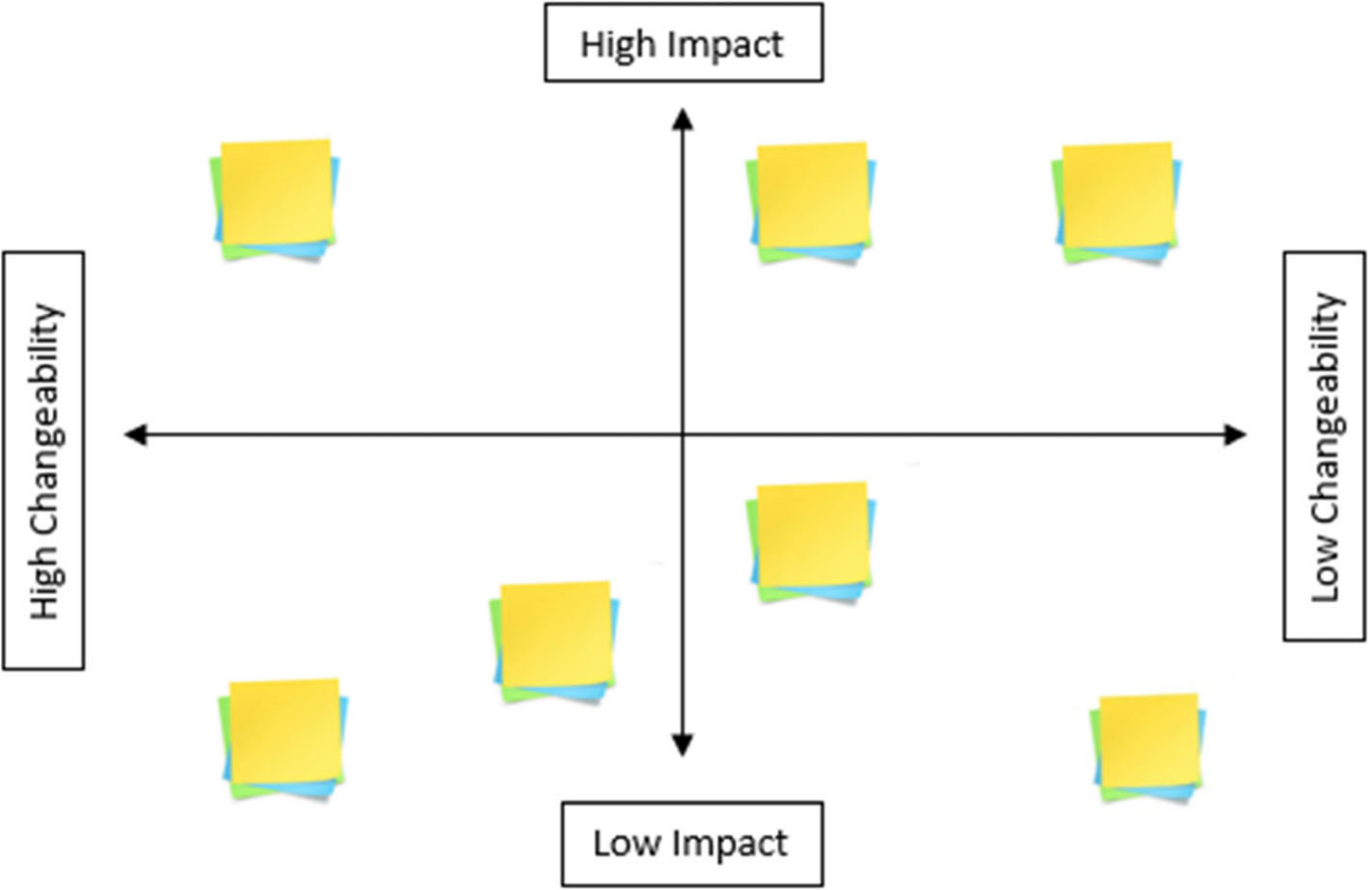
Grid to measure impact and changeability. The remaining themes are then placed by the assembled group in a grid per their level of impact and changeability

- Phase one—Download: The first phase involves summarizing the salient findings from formative research, which is done by listing the important points from existing knowledge and the formative research findings on index cards.
- Phase two—Cluster: These are then put on the wall for consideration. The findings are clustered together by the entire team per a common element and then appropriately named as a ‘theme’.
- Phase three—Brainstorm: Numerous themes are typically generated, so an elimination test is performed to keep only the relevant and significant themes. The remaining themes are then placed by the assembled group in a grid per their level of impact and changeability (Fig. 4). The themes that have low-impact or low-changeability are immediately ruled out; only high-impact and high-changeability themes are considered further. The group uses the themes as guides to discuss ideas of how to prompt HH.
- Phase four—Build: In the next phase, these ideas were developed into insights—or central concepts—that would be able to support the intervention.
- Phase five—Perform: The insights are assessed on their ability to cause a sustainable change in behaviour and their likelihood to be successfully implemented; this results in additional clustering exercise. The most promising insights are selected, and the group further refines the focus.
- Phase six—Agree on insight: The group discusses how to link the insights together into a focal insight, which is an enlightening deep truth about the behaviour and its causes [6].
- Phase seven—Develop: Once linked together into an focal insight, intervention implementation ideas are discussed. From this discussion, the components of the intervention are developed.
- Phase eight—Agree on theory of change: A theory of change is devised and a creative brief is written to summarize the findings and highlight the behavioural insight that will serve as the core behaviour change principle behind the intervention.
- Phase nine—Write brief: A creative brief, which includes the focal insight, the various intervention implementation and components ideas, and the Theory of Change is written and given to the creative team.

In this study, the design workshop was held in the USA throughout February 2016. The team followed the phases as described above.

### Create

The creative brief is given to a special creative team to develop the intervention. In the Create stage, the focal insight is expanded into the suite of materials that make up the intervention. These materials should initiate the change mechanisms postulated in the theory of change.

## Results

We now present the substantive results from the steps just described, as they occurred in this project. The greatest emphasis is placed on describing the execution and reported findings and insights from the design process (in the Build step) and the translation of the insights into intervention components (Create step). It should be noted that the results for the systematic review and the formative research are presented in other papers; however, the salient findings for each are briefly discussed below [18, 19].

### ASSESS: Establish evidence base

The systematic literature review produced three major findings [18]. First, the most recent HH interventions predominantly use education, reminders, and feedback as behaviour change mechanisms; they tend to incorporate information about the negative consequences arising from missed HH opportunities, they compare individual’s and hospital unit’s HH behaviour to other individuals and units, and they all set goals for increased HHC rates. The second major finding was that recent HH interventions use relatively few behaviour change techniques. Finally, most recent studies indicate that their interventions are grounded in behaviour change theory, yet little explanation is provided as to how the intervention implementation activities lead to behaviour change. It became apparent that there was a divide between the behavioural frameworks cited by the studies and how those constructs were operationalized. The findings from the background review provided a broad basis of knowledge, but also identified areas in which further investigation was required.

A framing workshop was held in November 2016 in the USA with stakeholders and experts. The stakeholders included employees of the Project Funder. Nurses and Infectious Disease directors from local hospitals were present to provide insight and expertise on HH behaviour. The research team—primarily RA and MHS—were present to lead the framing workshop, to discuss the theories of behaviour change and to review the factors that influenced HH behaviour identified from the general survey of the literature. The literature review was presented to workshop participants as background information for consideration during intervention development; this presentation included a summary of the factors found in intervention studies that had been found to influence HH practice in hospitals. This became the core group that guided the rest of the research project. It was during the framing workshop that we agreed on the aim of the intervention and outlined the various constraints surrounding the intervention design. The workshop ended with a framing statement (Fig. 2). Consensus on the framing statement was achieved through discussion and wordsmithing during the workshop itself. The core group decided the target population should be nurses in acute care units in the US hospitals. As discussed previously, different types of HCWs have different HHC rates and respond differently to HH campaigns. As nurses are on the frontline of healthcare delivery, the core group decided to create an intervention tailored specifically to nurses. We chose to focus on hospitals in the USA because the Project Funder was based there and had planned to commercialise the intervention in the USA if proven to be successful. In addition, we chose acute care units for two reasons: (1) acute care units provide rapid, active, time-sensitive treatment to patients who have a severe injury or illness, an urgent medical condition, or are recovering from surgery; thus, with the primary purpose to improve the health of such serious cases, HH is extremely important, and (2) it was for this reason that most hospitals with the Project Funder’s electronic compliance monitoring (ECM) system had installed it in their acute care units. The aim of the intervention was decided to increase a hospital unit’s HHC rates by 50% over its baseline rates, which aligns with increases observed in other HH trials specific to nurses in hospitals [14]. Then, the group identified hypotheses to explore in the formative research, which included the following:

- *Adding salience*: Would making evident the link between increased HHC rates and reduced HAI rates be a motivator?
- *Adding value*: Could we associate practicing HH with other values?
- *Linking to identity*: Could practicing HH be associated with being a ‘good nurse’?
- *Ritualizing the practice*: Would it be possible to ritualize the practice of HH and make it special?
- *Habit formation*: How could we reduce behavioural performance cost?

It was decided that the formative research would focus on investigating professional roles, status affiliation, social norms, motivation, physical manipulation of the hospital unit, and habit formation.

#### BUILD: Formative research and workshop

The formative research sought to further assess the relevance and behavioural change potential of factors identified from the literature and discussed during the framing workshop. Using as a web-based survey administered online to 500 nurses throughout the USA, the formative research determined that performing HH and complying with the recommendations were most likely a function of a hospital management’s communication ‘openness’, perceived performance by peers, increased interactions with patients and other staff members, and the reduction in stress, busyness, and cognitive load associated with role performance [19]. Also, it was noted that nurses were more likely to practice HH: (a) after performing a perceived higher-risk task like dressing a patient’s wound as compared to performing a low-risk task such as taking vitals and (b) upon exiting a patient’s room as compared to entering a patient’s room [19].

**Fig. 5:**
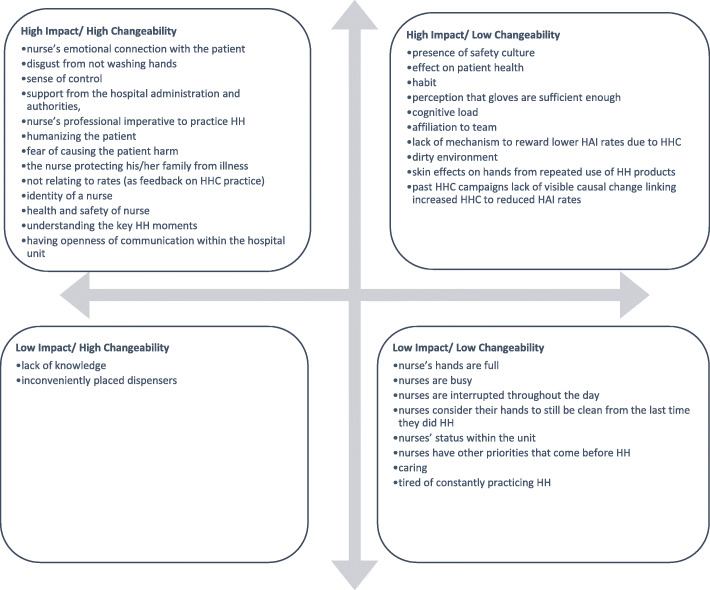
The impact and changeability of themes. The formative research findings were presented, then clustered into groups, and evaluated on their level of impact and changeability

Once the formative research had been analysed, a design workshop was held in the USA during February 2016 to develop a creative insight and brief. First, the formative research findings were presented. Workshop participants were then invited to write down as many factors that might influence HH as possible on Stickies. These were then clustered into groups collectively by the workshop participants and evaluated collectively on a matrix for their level of impact and changeability (Fig. 5). Examples from each of the categories have been described below for clarity:

- High impact and high changeability: From the formative research, we found that nurses feeling supported by hospital administration and authorities led to an increase in self-reported HH practice [19]. Thus, promoting a sense of support and unity is achievable and has the potential to lead to increased HHC rates.
- High impact and low changeability: HCWs often cite that using ABHR has negative effects on their hands (such as drying of the skin) [7]. It would neither be feasible nor in our area of expertise to create a new ABHR formula even if doing so would lead to increased usage.
- Low impact and high changeability: Changing a nurse’s lack of knowledge regarding HH could be easily changed by providing a form of education. However, educating nurses about the importance of HH does result in noticeable changes in HHC [18].
- Low impact and low changeability: Being busy, having their hands full, or having other pressing matters that need immediate attention all impact nurses’ HH behaviours [7]. However, these situations cannot be easily changed given the dynamics of the healthcare setting. In addition, while these are serious barriers to practicing HH, it could be argued that they are not the most consistent barriers. As such, our efforts are better spent focusing on factors that have high impact and high changeability.

**Fig. 6:**
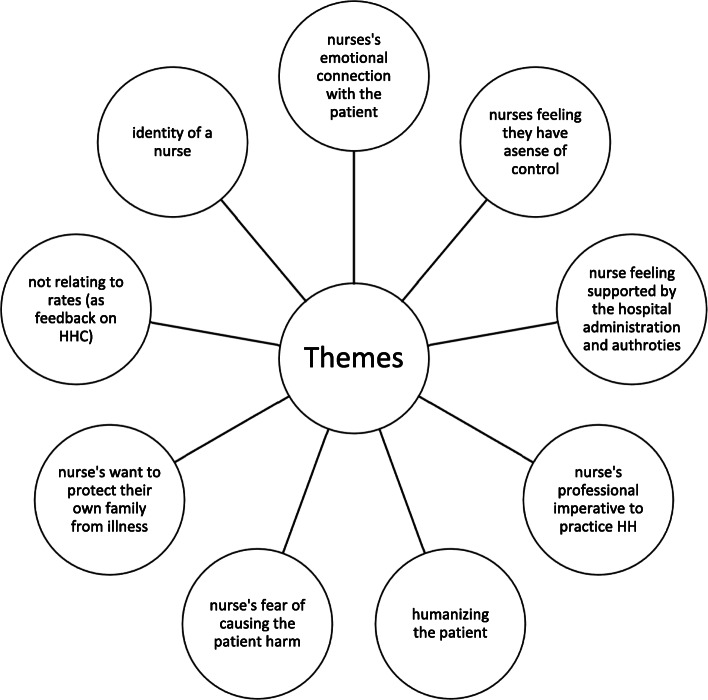
The most promising themes. The themes considered to be most impactful with the highest changeability were identified

The themes considered to be most impactful and with the highest changeability were identified through group consensus—with two thirds of the group having to be in agreement (Fig. 6). The themes considered to be most impactful with the highest changeability were as follows:

- Nurse’s emotional connection with the patient
- Nurse feeling a sense of control
- Nurse feeling supported by hospital administration and authorities
- Nurse’s professional imperative to practice HH
- Humanizing the patient
- Nurse’s fear of causing the patient harm
- Nurse’s want to protect their own family from illness
- Not relating to rates (need better feedback regarding HHC)
- Identity of a nurse

To further help identify a key insight, the themes were collapsed and combined into four insights associated with different types of behavioural determinants (as established by BCD): executive control, motives, social environment, and behaviour settings. Each of these insights are explained in detail in the following.

#### Executive control

Executive control is a broad term that describes higher-order cognitive processes such as memory, planning, problem solving, multi-tasking, inhibition, mental flexibility, and verbal reasoning [20]. The themes relating to ‘sense of control’ and ‘identity’ were placed under this term.

##### Sense of Control

Best practice care routines can easily be disrupted in acute care units resulting in relatively manageable and orderly shifts becoming chaotic and unruly. The workflow of nursing care delivery is constantly changing. During a shift filled with unpredictability, we speculate that nurses can gain a sense of control by practicing HH. The act of HH itself does not depend on others in the unit, and it has a substantial positive effect on patient outcomes. Thus, we predict that practicing HH gives nurses a sense of control where otherwise there is none.

##### Identity

In terms of identity, individuals are thought to be more likely to perform a behaviour that reflects the beliefs they have about themselves [21–23]. Self-identity and nested beliefs can change from engaging with a behaviour. Thus, it is hypothesised that a nurse who practices HH regularly can develop the identity or self-representation of being a good and diligent nurse.

#### Motives

Motives are evolved psychological mechanisms that lead to goal-directed behaviour [16, 24]. Performing a behaviour that produces a satisfactory outcome creates a rewarding experience, which prompts the individual to repeat the rewarding behaviour. Motives can be used to instigate behaviour change by modifying the target behaviour’s value. In the case of HH, relevant and emotional messages that tie the behaviour to patient outcomes, family values, and the role of a good nurse are hypothesised to motivate nurses to perform HH. Attaching motives and rewards to the performance of a target behaviour can lead to the establishment of new behavioural patterns. Two motives that could potentially be linked to HH are disgust and nurture.

##### Disgust

This motive evolved to facilitate disease-avoidance behaviour, thus protecting individuals against contamination. Disgust of contamination is an important driver of hygiene behaviour and has been harnessed to increase handwashing in various interventions [16, 25]. From the literature reviews conducted in the Assess step, we found that disgust of contamination was an important driver of hygiene behaviour and has been harnessed to increase handwashing in various interventions [16, 25]. In fact, other researchers have specifically studied disgust and dirt as key drivers in nurses’ infection control behaviours [26, 27]. Disgust can motivate nurses to practice HH for the obvious reason of reducing the nurses’ own perceptions of personal risk. As nurses are surrounded by disease and engage with people who are sick, practicing HH is speculated to be a way to make what would be perceived as a disgusting incident during the work day less disagreeable.

##### Nurture

Nurture drives caring and protective behaviours, and it attempts to influence the social world in favour of one’s in-group or kin. From the formative research conducted, we identified ‘other-oriented values’ as significantly important to nurses; these values encompass the nurse’s actions on behalf of the patient’s well-being and the interactions with patients in providing care, which could be considered nurturing. This motive can influence the practice of HH in two different ways. First, practicing HH is a way to protect one’s own family or immediate community from communicable diseases. We hypothesise that nurses are motivated to wash their hands to safeguard hospital pathogens from being introduced into their own homes. Second, patients are people and by practicing HH the nurse is taking care of the person. By not practicing HH, the patient is put at risk. Thus, we further hypothesise that humanising the patient allows for the nurture connection to be made.

#### Social environment

A major element of the social environment of a hospital is its ‘culture of safety’, which encompasses four main features: (a) acknowledgement of the high-risk nature of the hospital’s activities and the determination to achieve consistently safe operations, (b) a blame-free environment where individuals are able to report errors or near misses without fear of reprimand or punishment, (c) encouragement of collaboration across ranks and disciplines to seek solutions to patient safety problems, and (d) hospital commitment of resources to address safety concerns [28, 29]. Two key components that can be used to increase the performance of HH are communication openness between all HCWs in the hospital unit and direct feedback from administration and supervisors. Institutional support that includes positive and constructive feedback can also accentuate the importance and necessity of practicing HH.

#### Behaviour settings

Behaviour is also a function of the setting within which it takes place. The behaviour settings concept explains the relationship between individuals and the environment—both physical and social [30]. Behaviour settings are situations where people have learned what to expect from the environment and from other people’s behaviours. Each setting has a purpose, a designated place, a set of objects, and a prescribed set of behaviours. Each person entering a setting expects others, who are also contemporaneous participants, to perform their (implicitly) designated roles. A sustainable way of changing HH behaviour is by changing some element of its behaviour setting. In this case, role and norms are relevant aspects.

##### Role

Safeguarding patients is a professional imperative of nursing. By reemphasizing the role of nursing and what it entails, connecting the performance of HH to positive patient outcomes can possibly highlight how practicing HH is a vital part of being a nurse.

##### Norms

By making HH performance imperative, there is a drive to practice HH. We hypothesise that by emphasising the notion that others care and are watching to see if HH is performed will prompt nurses to be more aware of practicing HH.

These various insights were then linked together through facilitated engagement with the workshop members, resulting in the focal insight:

It’s under my control to reactivate [my commitment to] my professional code [of conduct] by caring for patients as persons via HHC to produce good patient outcomes and personal satisfaction.

**Fig. 7:**
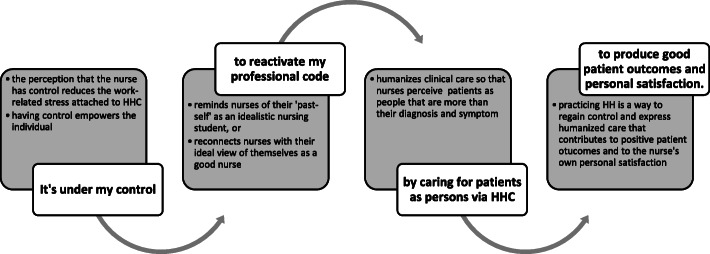
Explication of the focal insight

**Fig. 8:**
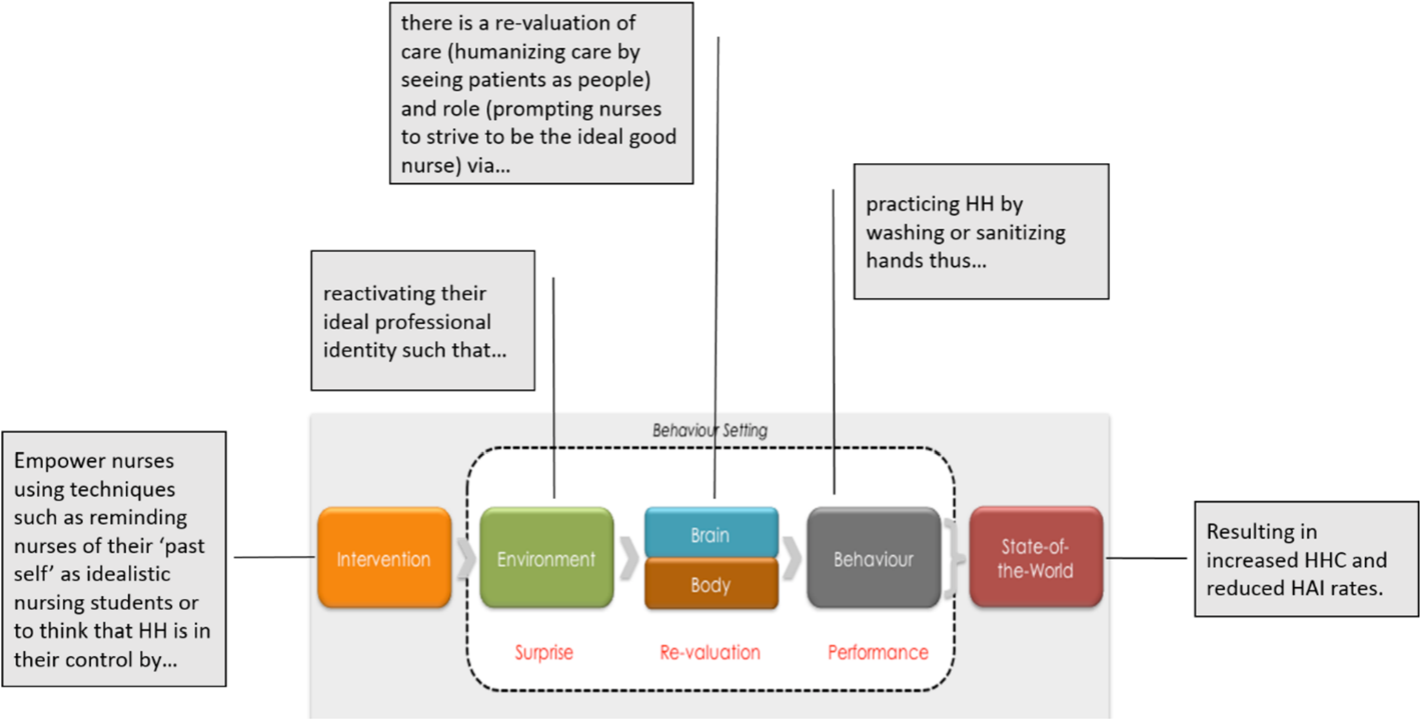
Focal insight translated into a theory of change. The focal insight was inked linguistically into a theory of change

**Fig. 9:**
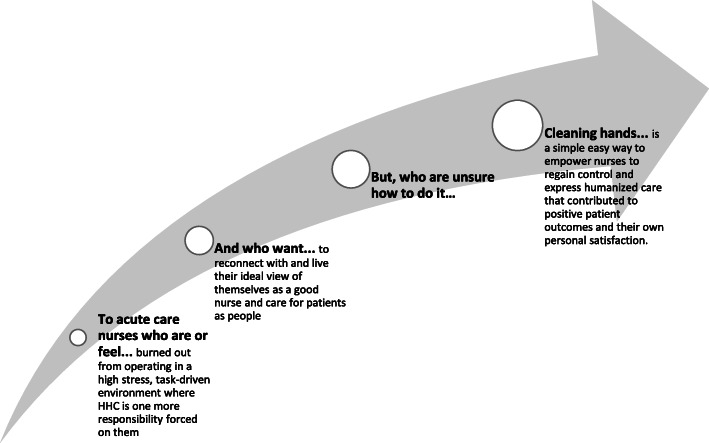
Focal insight translated into creative language. The brief, aiming to provide a succinct overview of the focal insight and strategy, rephrased the insight to help the creative team understand and address the challenge

This insight provided a single conceptual framework within which the intervention could be further developed. Essentially, nurses can be prompted to see HH as an opportunity to redefine their perceptions of patients as people to whom they are duty-bound to receive their care and protection. We postulate that by consistently practicing HH, nurses can rediscover the meaning and purpose of their role as a nurse and thus a caregiver—it is something good that nurses can do for themselves, their families and immediate communities, and their patients. In the process of cleaning their hands, nurses will also feel good because they are living up to their ideal nurse-self. The explication of the focal insight is provided in Fig. 7. The focal insight was then linked linguistically into a theory of change (Fig. 8) and subsequently translated into a creative brief. The brief, aiming to provide a succinct overview of the focal insight and strategy, rephrased the insight to help the creative team understand and address the challenge (Fig. 9).

### CREATE: Creative process

In response to this brief, the creative team (in this case, several marketers internal to GOJO Industries, a health and hygiene company) produced a simple intervention, called the *Mainspring Intervention*, that concentrated on a single approach: the threat to professional identity from non-compliance. Given the tight project budget, the short timeline for project completion, and the various constraints posed by hospitals—such as hospital regulations against altering the units or the inability to ‘pull nurses off the floor’ for a considerable amount of time—the creative team decided that a simple intervention would be easier to implement, would be less resource-intensive, and would allow for easier evaluation. This paper used the Template for Intervention Description and Replication (TIDieR) Checklist to ensure complete description of the intervention (Supplement 1).

The intervention was field-tested twice by the Project Funder using focus groups of practicing nurses (3 nurses per focus group). The intervention was delivered to the nurses individually, and then upon completion of the intervention, the nurses provided feedback in a focus group setting regarding delivery, presentation, and the message itself. Refinements to the intervention centred on wording and tone of the material being presented. Since the message regarding HHC could make participants uncomfortable, we included an exercise beforehand to reduce defensiveness and increase openness. This was introduced because a first focus group trialing the intervention had felt offended and became defensive when reading the HH message. The wording of the intervention’s message was slightly revised and delivered to a second focus group, which found it satisfactory and engaging after having been exposed to the self-affirmation exercise.

#### Mechanisms of the intervention

The first part of the intervention sought to reduce defensiveness using a values exercise, which was derived from self-affirmation theory. By reducing defensiveness, we hypothesised that nurses would be more open to receiving a message that challenged their professional identity and threatened their self-integrity. The message created awareness of a deficiency in HH behaviour but then provided constructive coaching by suggesting how to correct it. We posited that after the message was received, nurses would be motivated to achieve their professional best by performing HH more frequently at room entry. To ensure that this intention was translated into action, the intervention employed the implementation intention strategy to link the behaviour to a cue in the environment. This cue-behaviour link would theoretically elicit an automatic response.

#### Description of the intervention

The revised intervention consisted of three major parts: a self-affirmation exercise to reduce defensiveness, a message that challenged nurses’ perceptions about their HH practice, and an implementation intention activity. The self-affirmation exercise was a brief writing task that asked nurses to answer questions about values important to them. The message about HH introduced evidence that nurses were less likely to perform HH at room entry than at room exit, suggesting that nurses could improve their HHC by focusing on ‘foaming-in’ when entering a room. The implementation intention exercise prompted nurses to identify various features of the physical environment encountered regularly at room entry that could serve as cues to perform the target behaviour. This feature was used in the expressed implementation intention: ‘When I see [object], I will think ‘foam in!”

#### Theoretical underpinnings of the intervention

The behaviour change mechanisms were derived from two theories: self-affirmation [31] and implementation intentions [32–35].

##### Self-Affirmation Theory

Threating health information can sometimes produce defensiveness and resistance against the threat [36]. Self-affirmation theory proposes that individuals are motivated by a desire to maintain one’s worth and self-image as moral, adaptive, and capable [31, 37, 38]. Threatening health information creates dissonance with this image, which results in defensive responses as individuals seek to protect their self-integrity. To restore the integrity of the self, individuals may deny the potential risk and refuse to perform the adaptive behaviour. Potential opportunities for learning and growth are thus missed.

However, self-affirmation has been shown to reduce defensive processing of health risk information [36, 39–42]. Affirming the self before receiving threatening health messages reduces bias, promotes increased acceptance of the personal relevance of the message, and can affect risk perceptions over a short-term.

In this intervention, self-affirmation took the form of having participants write about self-defining values, which helped individuals protect their self-integrity and self-worth through the affirmation of alternative sources of self-identity and by reminding people what is important to them. Self-affirmation interventions have been shown to successfully influence a number of health-promoting behaviours [41].

##### Implementation intention strategy

This theory is a strategy that links intentions to the desired goal-directed behaviour and subsequently to the attainment of those goals [32–35]. Implementation intentions are specific, concrete plans phrased in the following manner: ‘When situation X rises, I will perform response Y.’ Thus, future critical situations are linked explicitly to goal-directed responses; when predefined situational cues are encountered, a goal-directed response occurs automatically. The intention-to-behaviour process works in the following way: an individual forms a plan that involves a specific situation—the ‘if’ part of the statement. This situation then becomes mentally represented. When the situation arises, the chosen goal-directed behaviour—the ‘then’ part of the plan—will be performed automatically and without conscious effort. Such automatization of behaviour in response to this cue removes deliberation on the part of the individual. Cognitive resources are made available for other mental process tasks while also avoiding goal-threatening or competing goals. Implementation intentions have been widely used in health promotion interventions and initiatives. They are among the best predictors of behaviour and behaviour change [43–45].

Taken together, the use of these mechanisms can be considered an example of a ‘wise’ intervention, which are psychologically precise interventions with brief implementations that aim is to alter self-reinforcing processes [46]. These seek to alter the psychological process that has developed over time and allow for the recurrent behaviour. Wise interventions are most likely to cause long-term gains in inherently recursive contexts in which positive experiences facilitate later positive outcomes [46].

#### Behavioural change techniques

**Fig. 10:**
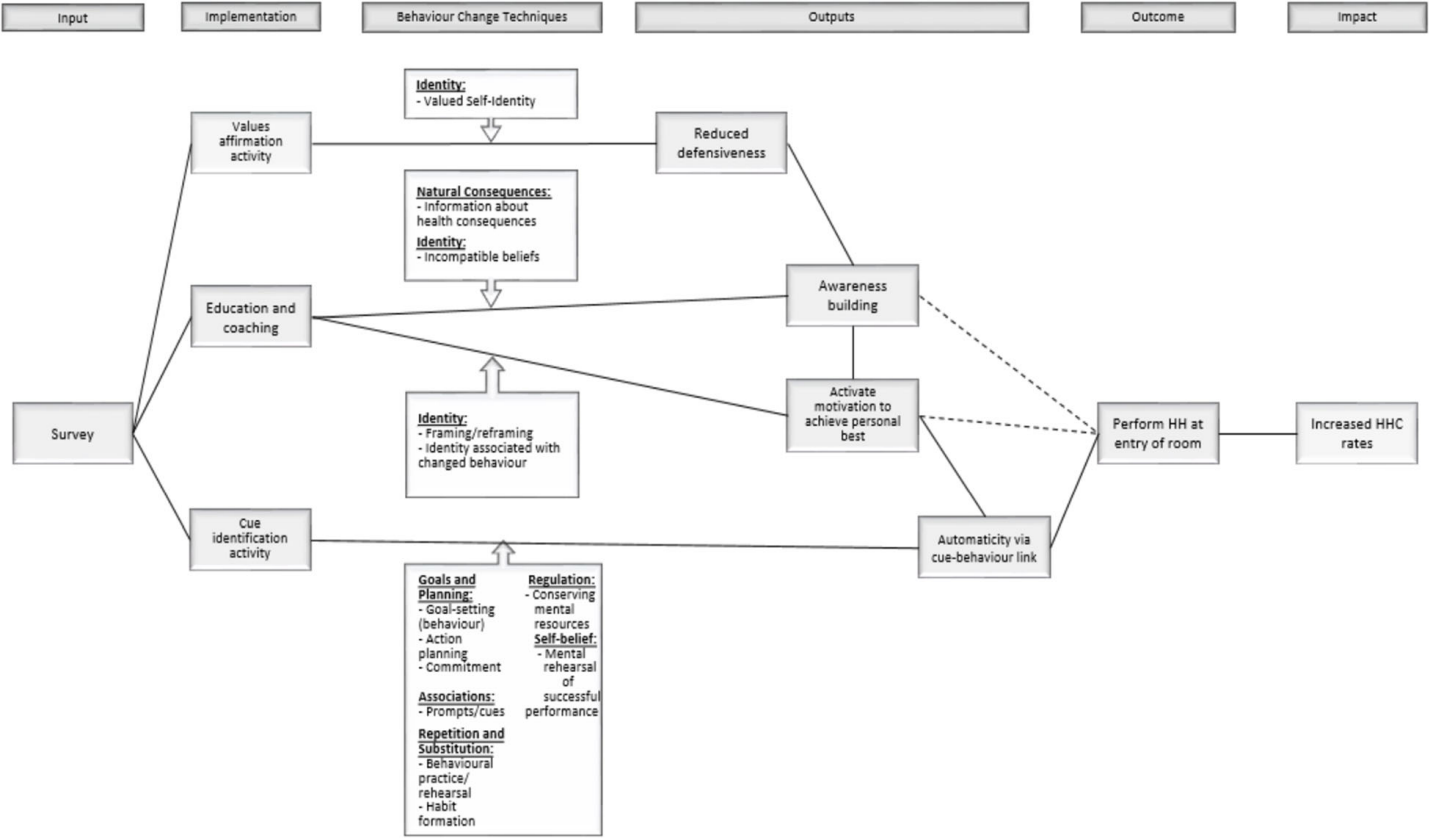
Mechanisms of change and their corresponding behaviour change techniques. Thirteen BCTs were utilized. Techniques were taken from across seven different categories of technique, including goals and planning, natural consequences, associations, repetition and substitution, regulation, identity, and self-belief

We used Michie et al.’s (2013) taxonomy of behaviour change techniques (BCTs) to define how our intervention’s theory of change was hypothesised to work via this ‘wise’ intervention structure [17, 47]. The authors inferred from formulation of the theory of change and literature on ‘wise’ interventions that thirteen BCTs could be inferred to underly the chosen intervention. Techniques were taken from across seven different categories of technique, including goals and planning, natural consequences, associations, repetition and substitution, regulation, identity, and self-belief (Fig. 10). As the intervention centres on the use of threat to professional identity, most BCTs fell within the identity category. In the values affirmation exercise, nurses were asked to write about cherished values as a means of affirming their identity (BCT 13.4). Then, the messaging or educational component raised awareness of the discrepancy in nurses’ HH practices when entering and exiting a patient’s room. Information about the health consequences of not practicing HH upon entry were emphasized (BCT 5.1). The health message drew attention to the incongruity between the nurses’ current HH practice and the required practice and sought to reframe the behaviour as being a fundamental component of nurse professionalism and code of conduct (BCT 13.3). This discomfort sought to prompt nurses to feel motivated to achieve their personal best. Practicing HH before entering a patient’s room would reaffirm their identity by reducing the cognitive dissonance between their ideal self-image and their day-to-day practice as a nurse (BCT 13.5). The cue-linking activity followed to help the nurses to explicitly identify the goal of practicing HH before entry and to create an action plan (BCTs 1.1 and 1.4). Nurses were asked to think of practicing HH and the environment near the patient’s room (BCT 15.2). The action plan had nurses link practicing HH to a cue in the environment that would lead to automaticity (BCTs 7.1 and 15.2). Making the behaviour automatic would reduce the deliberation and hesitation to perform HH thereby conserving mental resources (BCT 11.3). Afterwards, nurses were encouraged to say to themselves ‘As soon as I see [insert name of object] I will tell myself ‘clean your hands!” (BCT 1.9). The intervention ended by asking nurses over the next several days to use the object they selected as a reminder to clean their hands (BCTs 8.1 and 8.3).

#### Intervention materials and proposed delivery

The intervention is presented to participants in two separate parts in one day. The intervention is a self-guided activity and takes less than thirty minutes to complete. It is divided into two sections: the first part is the values affirmation activity and the second is the HH messaging with the implementation cue activity. Participants must complete the affirmation activity before being presented with the HH messaging.

Given the constraints of ‘taking nurses off the floor’ to participate, the intervention can be administered either in-person in the hospital unit or online. How the intervention is administered is at the hospital administration’s discretion. For the in-person delivery, the two parts of the survey are presented on separate sheets of paper. Respondents only receive the second page from the facilitator dependent on the completion of the values affirmation on the first page. When administered online, respondents complete the first exercise before being allowed to continue to the following activity. The intervention materials are provided in Supplement 2.

The facilitator oversees the delivery of the intervention in-person and ensures that the procedures are adhered to. The prompts for the facilitator are provided in Supplement 3. The facilitator does not need expertise or background in the topic of HH, and minimal training is required for the delivery of the intervention.

## Discussion

The HH behaviour and compliance of HCWs have been extensively studied. Even with the considerable amount of literature dedicated to this topic, very few studies are grounded in behaviour change theories [48]. The mainspring intervention was created using the BCD framework and was underpinned by its theory of change that utilised BCTS. In addition, this was one of the first studies to clearly describe how specific behavioural construct were applied to inform the development of intervention strategies. Thus, not only did the study used a cohesive approach to designing and creating an intervention, but the final product was the creation of an original HH behaviour change intervention. To our knowledge, a ‘wise’ intervention has not previously been used to improve HCW HH behaviour.

### The BCD approach to design

The BCD approach incorporates process steps that are rooted in design thinking for how to create an intervention. These creative processes emphasize the need to identify a central insight that can form the foundation for development of executional materials. Many frameworks provide steps on how to deduce themes from prior knowledge and formative research findings. Translating these themes into intervention components typically involves deductively linking behavioural determinants to behaviour change techniques, which are then used as stimuli for message formulation [49–51]. The BCD approach provides an inductive method for developing a central insight about the context of behaviour which is ‘built up’ through multiple brainstorming phases, including input and participation from a wide variety of sources. This process allows interventions to be designed through an iterative collaborative effort between the target population and the intervention designers [16].

The BCD approach is also flexible. In this case study, the process for developing the intervention deviated in several ways from the normal BCD process. The first deviation was seen in the formative research stage. BCD champions the use of a variety of data collection methods, specifically methods that are ‘near’—situationally and psychologically—to the behaviour that the intervention is trying to change. Such methods include observation or imaginative techniques for drawing informants into a virtual experience. This project only used a web-based survey to learn about the target population and the target behaviour due to time, resources, and budget constraints. The findings from formative research were based on the literature reviews and the survey and therefore were limited in comparison to fieldwork. As such, the development of the intervention relied heavily on the design workshop. In turn, the design workshop depended almost entirely on experts in the healthcare field (such as active and inactive nurses and those who were company employees with ties to healthcare).

A second deviation from standard practice occurred in the Create step. BCD stresses the importance of using a creative agency, often with several reverts to refine the creative direction and to build out the intervention itself. BCD heavily relies on the use of a creative agency as there is a lack of details about the actual development of the intervention (such as how to turn a creative brief into an intervention). Due to budget constraints, the project did not work with a creative agency, but rather used an in-house creative marketing team. However, given the constraints and restrictions, the in-house creative team did its best to faithfully translate the proposed components into an actual program with materials. The values affirmation exercise was included to reduce defensive processing of health risk information. It was also intended to guide nurses through a reflection on their own personal values and principles, which would then—it was hypothesised—lead into nurses considering their own professional code. By having nurses engage with internal discussions about values, the creative team assumed that nurses would receive the health message, be surprised, and in re-evaluating their behaviour would realise that practicing HH upon entering a patient’s room would be an easy way for them to realign with their professional code. By using cues to direct behaviour, we would help nurses translate intentions into actions, thus allowing them to take simple actions that would produce good patient outcomes and would therefore lead to their own personal satisfaction. The creative team included the intended components, although the messaging of trying to have nurses reactive their commitment to the professional code by caring for patients via HHC to produce good patient outcomes was not as overt as we had expected it to be.

Even though our design and create processes diverged from the usual BCD processes, the approach allowed for such adaptability to occur. The framework was shown to be able to accommodate different techniques and approaches so long as the main principles of each step were adhered to.

### The intervention

In this project, the entire development process was grounded in theory from the BCD design approach. BCD is founded in both behavioural science and design thinking practice and is based on a number of fundamental theories such as reinforcement learning, role theory, behaviour settings, and evolutionary psychology [16]. The intervention itself was underpinned by self-affirmation theory and intention implementation strategy. In addition, the behaviour change techniques in the intervention were pre-identified. Thus, intervention development has been grounded in theory from inception to development and has specifically described the mechanisms of change behind its theory of change. Essentially, the insight that hand hygiene could be improved by re-invigorating nurses’ professional identity as carers was implemented by a couched threat to that identity in the form of data which suggested nurses like themselves were more likely to protect themselves than their patients (i.e., ‘foam out’ but not ‘foam in’), but coupled with a self-affirmation exercise that would hopefully provide belief and motivation that improvement was possible.

Another distinct feature of the intervention was the use of the values affirmation activity and implementation intention exercise in the context of a HH intervention. The values’ affirmation activity has mainly been employed in educational settings to reduce the achievement gaps faced by minority students and women in science, technology, engineering, and mathematics courses [52–55]. Implementation intention exercises have previously been used in a wide-variety of health contexts ranging from promoting exercise [43] to prompting people to eat more fruit, but have been underutilized in changing the HH behaviour of HCWs [56–58].

Related ideas, such as personalised feedback and action planning, have been previously used in hand hygiene interventions (e.g. [59],), but these are much more personnel and time intensive. Such interventions can be tailored to reflect an individual’s particular circumstances and level of interest in behaviour change. Such tailoring can be effective [60–62]. However, the Project Funder was interested in producing an intervention that could be implemented with minimum expertise and effort in many different kinds of hospitals. The present intervention consisted entirely of a 5-min reading-and-writing exercise to change nurses’ cognitive processes directly. The activities encouraged the nurses to respond to ongoing, unpredictable work experiences in more adaptive ways to strengthen their professional identities independently. Most interventions focus on introducing a new experience to people’s lives. The change that occurs to the psychology of the person is indirect. Moreover, if the cornerstone of the intervention is introducing a new experience, the intervention can be vulnerable if that experience changes. This intervention encouraged nurses to see themselves as being in control of their own professional identity through the repeated practice of HH, rather than just relying on a specific experience to induce and then sustain change.

## Conclusion

Hand hygiene is widely accepted as the most important measure for the prevention of HAIs, but HHC rates are typically low. Numerous efforts have been made to increase HH among HCWs, and yet these initiatives have been unable to bring about sustained changes in behaviour. This paper detailed the creation of an original HH intervention that used the BCD approach, and we discussed the intervention design process, starting from the identification of the evidence base to the creation of the final intervention materials. What emerged from the development process was a ‘wise’ intervention, a simple intervention based on a specific psychological theory. The mechanisms, and the corresponding BCTs, behind the hypothesised Theory of Change were identified and explained, demonstrating how the constructs of the behavioural framework were operationalised. The intervention designed was relatively simple compared to most HH initiatives in the literature, both in terms of having relatively few components and relatively easy field implementation. This intervention will allow us to test: (a) how specific psychological processes contribute to the problem of low HH rates, (b) how our proposed intervention may change these processes in the hospital setting, and (c) how the expected change in nurses’ cognition transforms over time as a result of the intervention. Being so specific about how the intervention works, and basing the theory of change on strong theoretical and empirical grounds, should increase the likelihood of it being effective at sustainably increasing nurses’ HHC.

### Adherence to reporting guidelines

The TIDieR (Template for Intervention Description and Replication) Checklist was used to ensure that the original intervention discussed in this paper was described in sufficient detail (Supplement 1).

## Supplementary Information

**Additional file 1.** TIDieR (Template for Intervention Description and Replication) Checklist.

**Additional file 2.** Intervention Materials.

**Additional file 3.** TIDieR (Template for Intervention Description and Replication) Checklist: Delivery Protocol for Facilitator.

## Data Availability

The datasets during and/or analysed during the current study available from the corresponding author on reasonable request.

## Abbreviations

ABHR: Alcohol-based hand rubs
BCD: Behaviour Centred Design
BCT: Behaviour change technique
BCW: Behaviour change wheel
HAIs: Healthcare-associated infections
HCW: Healthcare worker
HH: Hand hygiene
HHC: Hand hygiene compliance
MRC: Medical Research Council
RANAS: Risks, Attitudes, Norms, Abilities, and Self-Regulation
TIDieR: Template for Intervention Description and Replication
