# Supplementary material for: Development of a behaviour change intervention using a theory-based approach, Behaviour Centred Design, to increase nurses’ hand hygiene compliance in the US hospitals
Source: Implement Sci Commun. 2021 Feb 18;2:23. doi: 10.1186/s43058-021-00124-x (PMC7893924; doi:10.1186/s43058-021-00124-x)
Supplement: Supplementary file 2 — Additional file 2. Intervention Materials. [file 43058_2021_124_MOESM2_ESM.docx]

Thank you for participating! We’d like to learn about values that are important to you. Please answer the following three questions about values.

1. Below is a list of values. We are interested to know which of these values are the most important to you in your everyday life – that is, not necessarily related to work, but important to you personally.

Write “1” next to your MOST IMPORTANT value.

Write “2” next to your SECOND MOST IMPORTANT value.

Write “3” next to your THIRD MOST IMPORTANT value.

_____ Creativity

_____ Courage

_____ Friendship

_____ Honesty

_____ Humor

_____ Justice

_____ Modesty

_____ Respect

_____ Spirituality

_____ Spontaneity

2. Please think about the value you wrote “1” next to. Why is this value *personally*  important to you?

__________________________________________________________________________________________________________________________________________________________________________________________________________________________________________________________________________________________________________________________________________________________________________________________________________________________________________________________________

3. Please briefly describe a time in your life (not involving your job/work) when the value you wrote “1” next to was particularly important to you.

____________________________________________________________________________________________________________________________________________________________________________________________________________________________________________________________________________________________________________________________________________________________________________________________________________________________________________________________________________________________________________________________________________________________

Thank you for completing this questionnaire!

**HAND HYGIENE – ‘REMINDER’ PROJECT**: *Please read the information below about hand hygiene.*

Proper hand hygiene is one part of a nurse’s responsibilities to ensure patient safety. Nurses usually clean their hands after **leaving** a patient’s room. Doing so protects the nurse from germs acquired during patient interactions. However, research using advanced methods of observation shows that nurses are less likely to clean their hands when **entering** a patient’s room. This means that nurses’ hands often carry germs into the patient’s room. Thus, nurses are not doing as much to protect their patients from germs as they are doing to protect themselves.

This highlights an important opportunity to improve hand hygiene upon **entry** to patient rooms. That is, we now know that ‘entering patient rooms’ is a specific situation in which nurses can focus their attention and achieve a noticeable increase in hand hygiene. Nurses should strive to clean their hands more consistently every time they enter a patient room. It is possible that nurses can create mental reminders to help them think about cleaning their hands in this specific situation.

Here's what you can do…

Think about the things/objects in the environment near most patient rooms in your unit.

This might include a sign (e.g., a room number), a part of a door, a dispenser, etc.

Ideally, identify some object that doesn’t move – something that will be present every time you approach most patient rooms. Also, try to identify something distinctive – something with a shape, color, or size that will stand out and catch your attention each time you approach the room.

🡺 Please list the object you identified here: ___________________________________________

Next, make a plan involving the object you identified. Tell yourself, “As soon as I see [*insert name of object*] I will tell myself ‘clean your hands!’”

**Please fill in the blank in the statement below:**

🡺 **“As soon as I see ________________________________ I will tell myself ‘clean your hands!’”**

Over the next several days:

- Please remember the object you selected
- Whenever you see that object, please use that object as a reminder to clean your hands.

Thank you
