# Supplementary material for: Development of a behaviour change intervention using a theory-based approach, Behaviour Centred Design, to increase nurses’ hand hygiene compliance in the US hospitals
Source: Implement Sci Commun. 2021 Feb 18;2:23. doi: 10.1186/s43058-021-00124-x (PMC7893924; doi:10.1186/s43058-021-00124-x)
Supplement: Supplementary file 3 — Additional file 3. TIDieR (Template for Intervention Description and Replication) Checklist: Delivery Protocol for Facilitator. [file 43058_2021_124_MOESM3_ESM.docx]

***Mainspring Intervention***

*Directions for In-Person Delivery by Facilitator*

Black text – indicates materials nurses (research participants) will receive and complete

*Blue, italic text* – indicates descriptions of how/where the intervention will be delivered, instructions the facilitator will provide to the nurses, etc. Nurses will not see these sections in the intervention materials – these sections are just for the research team to consider, but will be removed from materials to be used in the hospitals.

*Setting for intervention:*

- *Ideally, the facilitator will meet with several nurses in a group and will have 5-10 min to deliver the intervention materials on paper.*
- *This will take place during nurses’ work day (during their shift) in each respective unit’s break room/ conference room.*
- *Recruitment will be the responsibility of the respective hospitals, units, and nurse mangers.*

*Introduction:*

- *We’ll give a brief description of who we are and what we’re asking nurses to do.*
- *Maybe something like this…*
  - *I’m a researcher [from XXX]. We’re partnering with the hospital to learn about hand hygiene.*
  - *In a minute I’ll give you some information on hand hygiene.*
  - *First, I’d like to ask you to complete a brief questionnaire. We’re trying to learn about values that are important to people in the healthcare field. Today, I’d like to ask your help answering a couple brief questions.*
  - *Materials we fill out today won’t be shared with anyone at the hospital.*

*Next, pass out a 1-page questionnaire containing the 3 questions below:*

Thank you for participating! Please answer the following three questions about values.

1. Below is a list of values. We are interested to know which of these values are the most important to you.

Write “1” next to your MOST IMPORTANT value.

Write “2” next to your SECOND MOST IMPORTANT value.

Write “3” next to your THIRD MOST IMPORTANT value.

_____ Creativity

_____ Courage

_____ Friendship

_____ Honesty

_____ Humor

_____ Justice

_____ Modesty

_____ Respect

_____ Spirituality

_____ Spontaneity

2. Please think about the value you wrote “1” next to. Why is this value *personally*  important to you?

__________________________________________________________________________________________________________________________________________________________________________________________________________________________________________________________________________________________________________________________________________________________________________________________________________________________________________________________________

3. Please briefly describe a time in your life (not involving your job/work) when the value you wrote “1” next to was particularly important to you.

____________________________________________________________________________________________________________________________________________________________________________________________________________________________________________________________________________________________________________________________________________________________________________________________________________________________________________________________________________________________________________________________________________________________

Thank you for completing this questionnaire!

*Next, we’ll pass out a page or two containing the information below…*

Please read the information below about hand hygiene.

Proper hand hygiene is one part of a nurse’s responsibilities to ensure patient safety. Nurses usually clean their hands after **leaving** a patient’s room. Doing so protects the nurse from germs acquired during patient interactions. However, research using advanced methods of observation shows that nurses are less likely to clean their hands when **entering** a patient’s room. This means that nurses’ hands often carry germs into the patient’s room. Thus, nurses are not doing as much to protect their patients from germs as they are doing to protect themselves.

This highlights an important opportunity to improve hand hygiene upon **entry** to patient rooms. That is, we now know that ‘entering patient rooms’ is a specific situation in which nurses can focus their attention and achieve a noticeable increase in hand hygiene. Nurses should strive to clean their hands more consistently every time they enter a patient room. It is possible that nurses can create mental reminders to help them think about cleaning their hands in this specific situation.

Here's what you can do…

Think about the things/objects in the environment near most patient rooms in your unit.

This might include a sign (e.g., a room number), a part of a door, a dispenser, etc.

Ideally, identify some object that doesn’t move – something that will be present every time you approach most patient rooms. Also, try to identify something distinctive – something with a shape, color, or size that will stand out and catch your attention each time you approach the room.

Please write the object you identified here: _____________________________________________

Next, make a plan involving the object you identified. Tell yourself, “As soon as I see [*insert name of object*] I will tell myself ‘clean your hands!’”

Please fill in the blank in the statement below:

“As soon as I see ________________________________ I will tell myself ‘clean your hands!’”

*To concludes the session, deliver the information below verbally after the nurses have completed the questionnaires.*

- *I’d like to ask you to do two things over the next several days:*
  - *(1) please remember the object that you selected*
  - *(2) whenever you see that object, please use the object as a reminder to clean your hands*
- *Thank nurses for their time*
